# Efficacy of Human-Induced Pluripotent Stem Cell-Derived Neural Progenitor Cell Replacement Therapy in a Vascular Dementia Animal Model

**DOI:** 10.1007/s13770-025-00706-z

**Published:** 2025-02-14

**Authors:** Jang Hun Kim, Ho-Young Kang, Jihun Lee, Jong-Hoon Kim, Dongho Geum, Dong-Hyuk Park

**Affiliations:** 1 Department of Neurosurgery, Korea University Anam Hospital, Korea University College of Medicine, Seoul, Republic of Korea; 2 Center of Innovative Cell Therapy and Research, Korea University Anam Hospital, Korea University College of Medicine, Seoul, Republic of Korea; 3 Laboratory of Stem Cells and Tissue Regeneration, Department of Biotechnology, College of Life Sciences and Biotechnology, Korea University, Seoul, Republic of Korea; 4 Department of Medical Science, College of Medicine, Korea University, Seoul, Republic of Korea

**Keywords:** Bilateral carotid artery stenosis, Cognitive impairment, Human-induced pluripotent stem cells, Neural progenitor cells, Vascular dementia

## Abstract

**Background:**

Cell replacement therapy is the only treatment that restores or repairs the function of impaired tissues in neurodegenerative diseases, including vascular dementia (VaD); however, current VaD treatments focus on slowing or mitigating the underlying small vessel disease progression. We aimed to verify the improvement in neurocognition after administering human-induced pluripotent stem cell (hiPSC)-derived neural progenitor cells (NPCs) from in a VaD animal model.

**Methods:**

After anesthesia, 10–12-week-old male C5BL/6 mice underwent sham or bilateral carotid artery stenosis (BCAS) surgeries. For BCAS, 0.18-mm micro-coils were wound around the bilateral common carotid arteries to induce chronic vascular insufficiency in the global brain. One day after surgery, the mice were administered phosphate buffer solution or NPC from hiPSCs via the tail vein for 15 d, and divided into sham (n = 6), VEH (n = 6), and NPC (n = 7) groups. Three months after the surgery, neurobehavioral tests including the Y-maze test (YMT), passive avoidance test (PAT), and novel object recognition test (NORT) were performed. Finally, mice brains were sectioned for evaluating microglia (Iba-1), astrocyte (GFAP) activation, and myelin (MBP) degeneration through immunohistochemistry (IHC).

**Results:**

PAT latency (*p* = 0.01) and discrimination index in the NORT (*p* = 0.043) increased considerably in the NPC group than in the VEH group. However, alterations in YMT were not considerably higher in the NPC group than in the VEH group (*p* = 0.65). IHC tests revealed that the GFAP- and IBA-1-positive cell number was remarkably lower in the NPC group than in the VEH group (*p* < 0.05). Moreover, MBP density was higher in the NPC group.

**Conclusion:**

hiPSC-derived NPCs have therapeutic potential in cerebral hypoperfusion VaD mice; it improves the working memory of VaD animals by diminishing inflammatory reactions and protecting them from demyelination.

**Supplementary Information:**

The online version contains supplementary material available at 10.1007/s13770-025-00706-z.

## Introduction

Dementia is an increasingly common diagnosis in the older population, and its incidence is expected to increase exponentially in the future [1]. Among the various subtypes of dementia, vascular dementia (VaD) is the second most common after Alzheimer’s disease (AD), and approximately 25% patients diagnosed with dementia are recognized as having VaD in Korean epidemiologic studies [2, 3]. Vascular dementia (VaD) is caused by cerebrovascular diseases that directly or indirectly damages the brain structures. It affects not only the cerebral cortex or hippocampus, but also whole brain vascular territories, including white matter (WM) lesions. Subcortical WM lesions are characteristically observed in human cerebrovascular diseases of VaD and believed to be responsible for cognitive and behavioral impairments [4].

The current treatment strategies for VaD focus on slowing or mitigating underlying small vessel disease progression and improving clinical symptoms [5]. Ongoing microinfarction can aggravate brain atrophy and cognitive impairment; therefore, antiplatelet agents or vasodilators, such as nimodipine, can be administered [6–8]. Additionally, cholinesterase inhibitors or cholinergic drugs, such as choline alfoscerate (ChA) can be used for targeting the basal forebrain cholinergic nuclei and degenerated cholinergic fibers [9, 10]. Sometimes, antidepressants such as selective serotonin reuptake inhibitors can be used for symptom relief and behavioral change mitigation [11]. However, their effectiveness is not yet fully established. Due to the lack of neuronal regeneration in older human brains, the above-mentioned drugs have limitations in treating disease pathophysiology or restoring damaged neural structures.

Instead, cell replacement therapy (including stem cell therapy) involves cell supplementation to restore or repair impaired tissue function in neurodegenerative diseases [12, 13]. The therapeutic potentials of various cell types, such as adipose tissue-derived mesenchymal stromal cells, human umbilical cord blood-derived mesenchymal stromal cells, human placenta amniotic membrane-derived mesenchymal stromal cells, and human-induced pluripotent stem cells (hiPSCs) have been demonstrated [14].

Among the various cell types, neural progenitor cells (NPCs) from hiPSCs are regarded as the nearest progenitor cell for human neuronal cells that can be acquired from induced stem cell originated from human dermal fibroblast [15], 16 Neural progenitor cells (NPCs) have several characteristic strengths: self-renewable, ability to generate many progenies, and ability to differentiate into principal central nervous system (CNS) cell types (such as neurons, astrocytes, and oligodendrocytes) [17–19]. Recent studies have demonstrated the promising role of NPCs in various neurodegenerative conditions due to their ability to modulate the local microenvironment through paracrine signaling.[20] This paracrine effect can lead to neuroprotection and repair by secreting bioactive factors that reduce inflammation and promote neuronal survival, even when direct cell engraftment is limited [21]. Moreover, hiPSC-derived NPCs offers a patient-specific approach, potentially minimizing immune rejection and enhancing therapeutic outcomes in VaD models [15]. Subcortical WM has the axonal fibers of neuron as well as multiple neuronal supportive cells such as Schwann cells or abundant extracellular matrix; therefore, hiPSC-derived NPCs can be potentially effective for restoring the damaged subcortical lesions in VaD models [16].

Here, we attempted to verify the improvement in neurocognition after administering hiPSC-derived NPCs in a VaD animal model. In particular, the animal model was prepared after bilateral carotid artery stenosis (BCAS) surgery by winding micro-coils, which was regarded as a novel VaD mouse model presenting subcortical WM ischemia with degenerated axonal fibers.

## Materials and methods

### Animals, BCAS surgery, and hiPSC-derived NPC administration

All experimental procedures were approved by the Institutional Animal Care and Use Committee of the Korea University (2017-0055). Animal care and handling were performed in accordance with the Guide for the Care and Use of Laboratory Animals of the National Institutes of Health and reported according to the Animal Research: Reporting of *In Vivo* Experiment (ARRIVE) guidelines. All surgeries were performed under isoflurane inhalant anesthesia and efforts were made to minimize suffering within the animal facility during the animal’s light cycle. All mice were housed in a climate-controlled room with a 14-h/10-h light/dark cycle and ad libitum access to food and water.

**Fig. 1:**
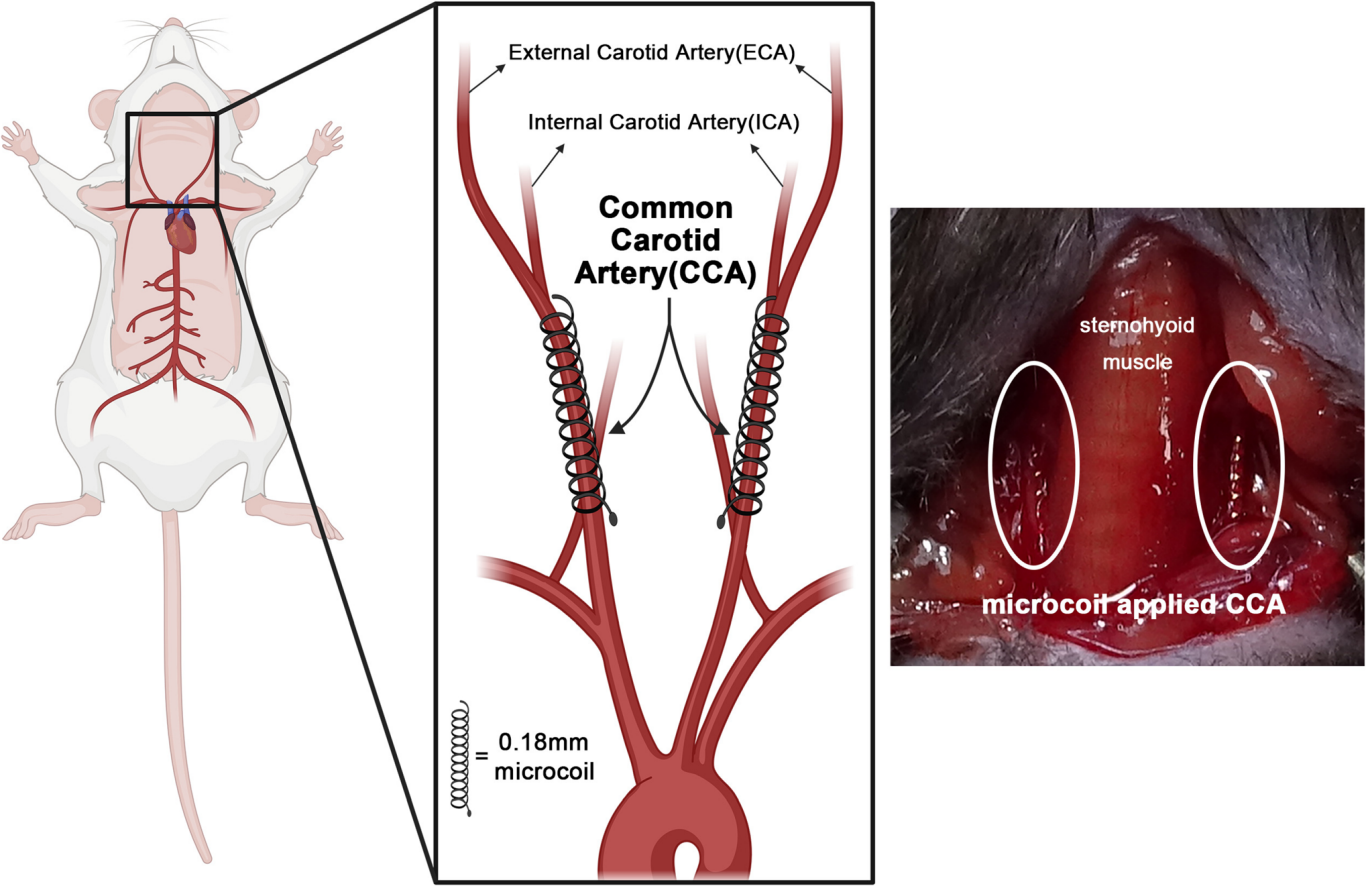
Schematic illustration of bilateral carotid artery stenosis (BCAS) surgery. This figure depicts the placement of micro-coils around the common carotid arteries to induce chronic cerebral hypoperfusion

We prepared 10–12-week-old male C5BL/6 mice before BCAS surgery [22, 23]. The mice were anesthetized with 5% isoflurane and maintained under anesthesia with 1.5% isoflurane in a 70% N_2_O and 30% O_2_ mixture to induce prolonged cerebral hypoperfusion and progressive ischemic attacks. As illustrated in Fig. 1, a midline incision was made to expose the common carotid arteries (CCAs), while avoiding damage to the esophagus, trachea, neck muscles, and vagus nerves. Two 4–0 black silk sutures were placed under the proximal and distal portions of the left CCA and used to gently lift the artery for placing the micro-coil (0.18 mm diameter; Waken B Technology Company Limited, Japan) by rotating it around the artery just below the carotid bifurcation. Subsequently, a second micro-coil was placed in the right CCA. The incision was then closed, and the animals were raised for three months. In the sham mice, every surgical procedure was performed as described above except for coil windings.

The mice were placed in a warm recovery cage immediately post-operatively and monitored until they were fully awake, mobile, and interested in food and water. Once fully awake, they were placed in their home cages in the animal facility and monitored daily for signs of sickness or discomfort, including infection at the incision site, lethargy, disinterest in food and water, weight loss, and seizures. The mice were euthanized by full inhalation of CO_2_ gas after the behavioral tests.

Mice were divided into three groups according to the drugs administered 24 h after surgery.

1. **Sham**: phosphate buffer solution (PBS) administration after sham surgery (n = 6)
2. **VEH**: PBS administration after BCAS (n = 6)
3. **NPC**: NPC after hiPSC administration following BCAS (n = 7).

**Fig. 2:**
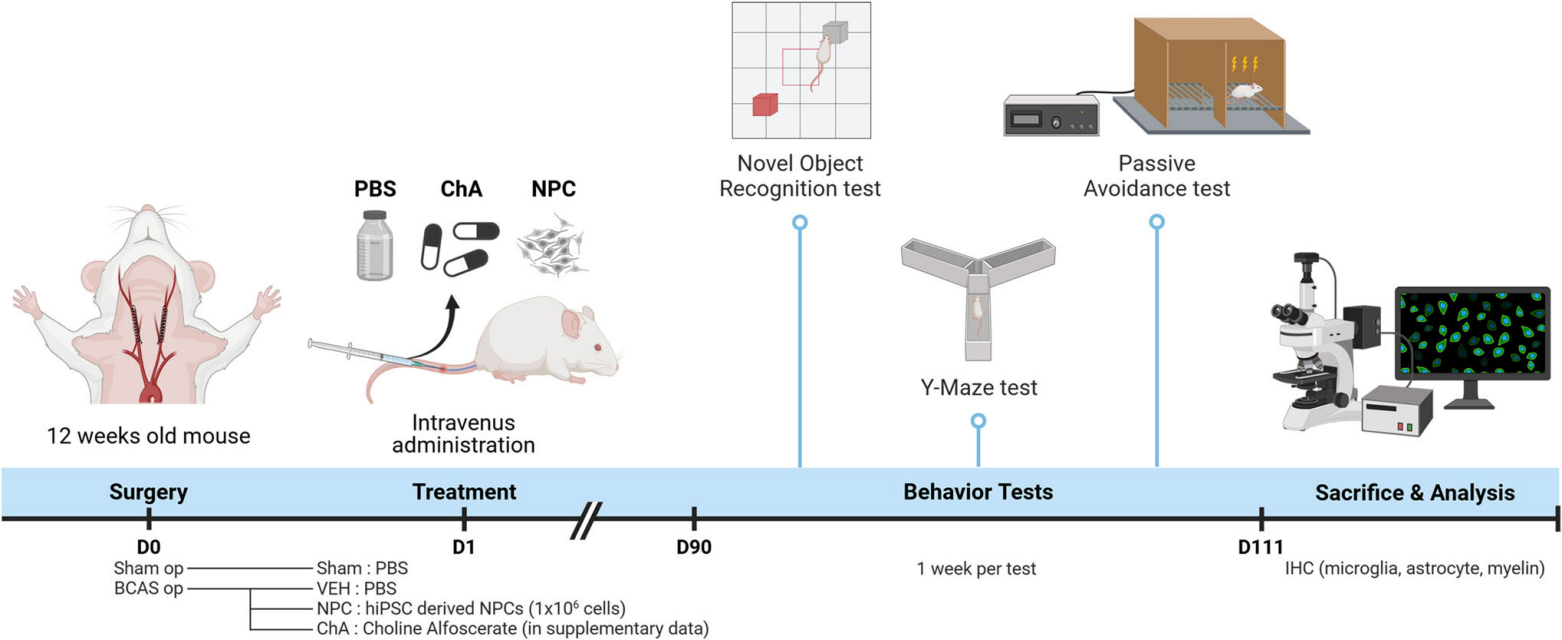
Schematic illustration of bilateral carotid artery stenosis (BCAS) surgery and experimental timeline. Mice aged 10–12 weeks underwent BCAS or sham surgery. Post-surgery, the mice were divided into three groups: Sham (n = 4), VEH (n = 5), and neural progenitor cell (NPC; n = 5). NPCs were administered intravenously 24 h post-surgery for 15 d. Neurobehavioral tests, including the novel object recognition test, Y-maze test, and passive avoidance test, were conducted over three months, with one test per week. Euthanasia and immunohistochemical analysis were performed to assess microglia, astrocytes, and myelin integrity

NPC from hiPSCs were purchased from the company (NEXEL Co., Ltd. Seoul, Korea) and injected through the tail vein for 15 d. The dosage was 300 µL (1 × 10^6^ cells). NPC production followed the progenitor neural cell production method of a preceding patent (10-2019-0071402), and the NPCs were differentiated from hiPSCs originating from human dermal fibroblasts. The experimental setup is shown in Fig. 2.

### Neurobehavioral tests

#### Y-maze test (YMT)

Spatial working memory and cognition were assessed using spontaneous alteration behavior in the YMT on post-operative day 90 [24]. The maze consisted of three 120° white plastic arms (35 cm long, 6 cm high, and 15 cm wide). The floor was cleaned using 10% ethanol to eliminate olfactory cues. Each mouse was positioned at the end of the start arm and allowed to move freely through the maze during an 8-min session. The movements of the mice were recorded using a video camera and the number of arms entered was automatically counted. Arm entry was considered complete when the hind paws were within the arm. The percentage of spontaneous alterations was calculated as follows: actual alteration (entry into all three arms on consecutive occasions) / (total number of arm entries − 2) × 100. A high percentage of spontaneous alterations is considered indicative of good spatial working memory and cognition.

#### Passive avoidance test (PAT)

Passive avoidance test (PAT) was performed 1 week after YMT to evaluate intermediate memory and learning abilities. Additionally, PAT was conducted as previously described (Sitechkorea, SITECH TECHNOLOGIES INC, Seoul, Korea) [25]. The test typically began by placing the mouse in a lit chamber for 10 s and then opening the guillotine door between chambers. The mice entered the dark chamber naturally because they were highly exploratory and preferred the dark chamber to the lit chamber. With the door closed, a single electric shock (0.8 mA, 2 s) was delivered through the grid floor. The mice remained in the dark chamber for another 10 s to strengthen the association between the properties of the chamber and the foot shock. The mice were then returned to their respective cages. After 24 h, the mice were re-placed in a lit chamber with open doors between the chambers and the time required to enter the dark chamber was also recorded. Normal mice were slow to enter the dark chamber and, often, did not enter at all up to a 300-s cutoff latency, presumably because they remembered that a shock had been delivered in the dark chamber the previous day.

#### Novel object recognition test (NORT)

The NORT was performed to determine the ability of a compound to increase or impair learning and short-term recognition memory. For habituation, mice were placed in an empty open-field box (40 × 40 × 40 cm^3^) and allowed to freely explore the open field for 15 min. On the familiarization day, two identical objects (A and B) were placed in the box and the mice were allowed to explore them for 10 min. The mouse was considered to have explored an object when its head was facing within 1 inch of the object. The mice were then removed from the box. After a 1 d of retention interval, the mouse was placed back in the box with two objects in the same location; one of the familiar objects was replaced by a novel object (A & C), and the mouse was allowed to explore the two objects for 5 min. Short-term recognition memory performance was described as the ratio of the time spent on a new object (N) minus the time spent on a familiar object (F) to the total time spent on both objects. Discrimination index was defined as: (N − F)/(N + F).

### Tissue preparation and immunohistochemistry (IHC)

After completing the behavioral test, the mice were anesthetized with intraperitoneal injections of 100 mg/kg ketamine and 10 mg/kg xylazine, perfused transcardially with PBS, and fixed in 4% paraformaldehyde in phosphate buffer (0.1 M). The brain was extracted without any damages and fixed in 4% paraformaldehyde solution overnight at 4 °C, and placed in a 30% sucrose solution for 4 d for cryoprotection. The cryoprotected brains were embedded in an optimal cutting temperature compound and frozen in dry, ice-cooled isopentane. The frozen brains were sliced into 20 µm coronal sections using a cryostat vibratome (CM3050S; Leica Microsystems, Wetzlar, Germany) and stored at − 80 °C until further processing.

For IHC, the tissue sections were treated with 2 N HCl for 30 min at 37 °C followed by neutralization with immersion in 0.1 M borate buffer (pH 8.5) for 10 min at room temperature. Non-specific binding was blocked by incubating the sections with 10% horse serum in PBS. The sections were incubated overnight with primary antibodies at 4 °C. The primary antibodies used for IHC were: rabbit polyclonal anti-ionized calcium-binding adapter 1 (Iba-1; 1:600; Osaka, Japan) to label the microglia, mouse monoclonal anti-glial fibrillary acidic protein (GFAP; 1:1000; Sigma-Aldrich, St. Louis, MO, USA) to label the astrocytes, and monoclonal anti-myelin basic protein (anti-MBP; 1:650; Millipore, Billerica, MA, USA) to label the myelin sheath [26]. The following secondary antibodies were used: Alexa Fluor 488-conjugated anti-mouse immunoglobulin G (IgG) (1:400; Invitrogen), Alexa Fluor 594-conjugated anti-goat IgG (1:800; Invitrogen), Alexa Fluor 488-conjugated anti-rabbit IgG (1:400; Invitrogen), and Rhodamine Red X-conjugated anti-mouse IgG (1:100; Jackson ImmunoResearch Inc., Burlingame, CA, USA). The sections were counterstained with DAPI (Thermo Fisher Scientific, Waltham, MA, USA) and visualized under a fluorescence microscope (Axio Scan.Z1; Carl Zeiss, Oberkochen, Germany). Immunofluorescence images were acquired using a Zeiss LSM 700 confocal laser microscope (Carlsbad, CA, USA), and antibody-positive cell counts and fluorescence density were measured.

### Quantification and statistics

For the quantifications of IHC results, six sections were obtained every 280 µm beginning with a section 1.2 mm rostral to the bregma. Iba-1- and GFAP-positive cells were counted under a fluorescence microscope in three microscopic fields of the cortex, hippocampus, and corpus callosum in each section. MBP-positive cell density was evaluated in images captured from the corpus callosum areas using MetaMorph imaging software (version 7.8.1; Molecular Devices, San Jose, CA, USA). The sum of the values from all six sections was used as the final value.

All data are presented as mean ± standard error of the mean. One-way ANOVA was conducted initially, and differences with *p* < 0.05 were deemed statistically significant. Post-hoc analysis was performed using Scheffe’s method.

## Results

### Neurobehavioral tests

**Fig. 3:**
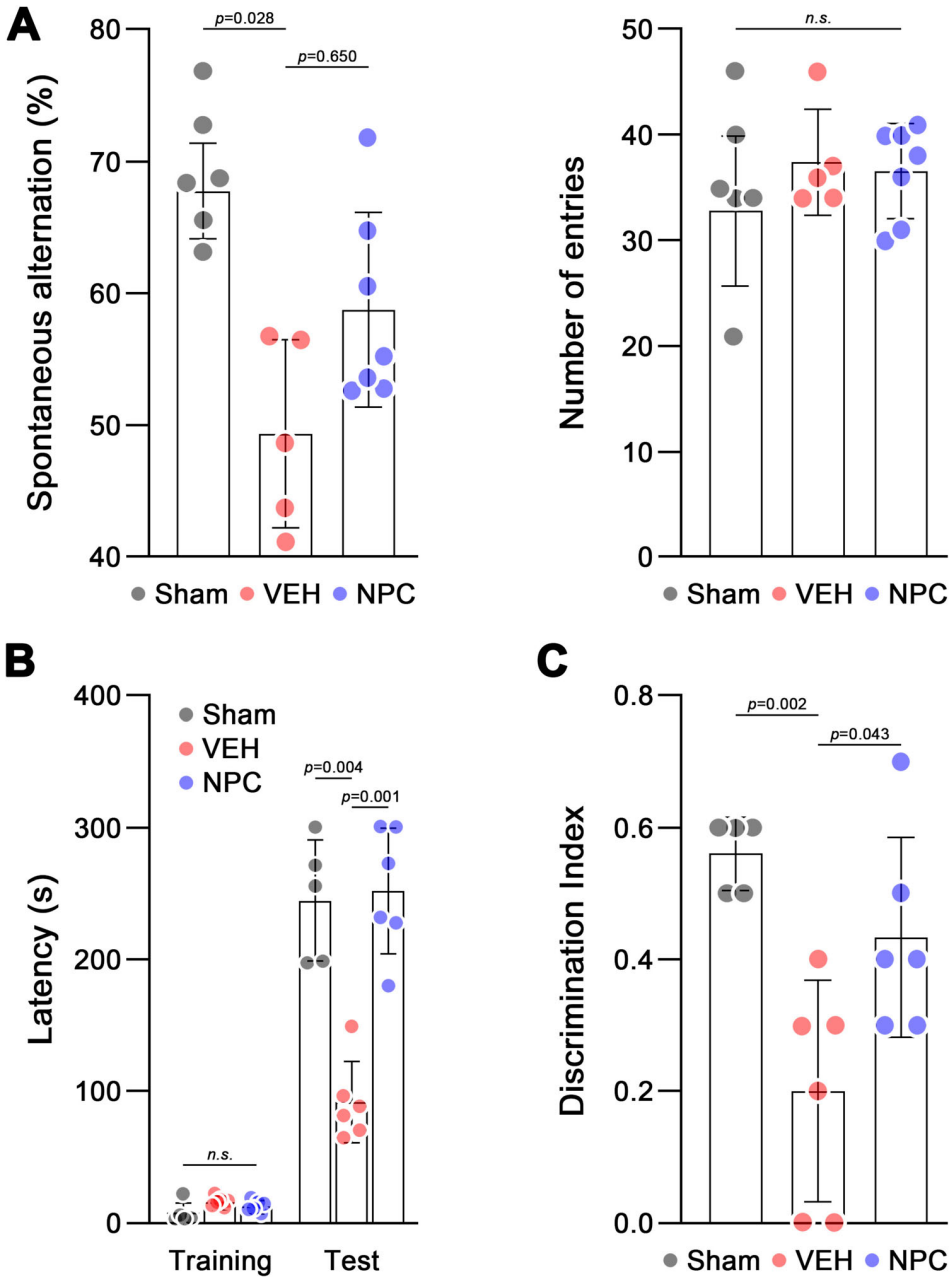
Results of neurobehavioral tests assessing cognitive function in mice. (A) Y-maze test (YMT) measuring spontaneous alternation percentage, indicating spatial working memory. (B) Passive avoidance test (PAT) showing latency times, reflecting learning and memory retention. (C) Novel object recognition test (NORT) displaying the discrimination index, assessing recognition memory. Data are presented for three groups: Sham, VEH, and NPC. Statistical significance is indicated by *p*-values, with n.s. denoting non-significant differences (*p* > 0.05). Each data point represents an individual mouse, with error bars showing mean ± SEM

In the YMT, the mice demonstrated non-specific differences in the “number of arms entered,” which refers to similar physical activities observed between the groups. In “Alteration,” the BCAS groups (VEH: 49.3 ± 3.2, NPC: 58.7 ± 2.8) showed relatively lower alteration rates than the sham group (70.0 ± 1.1), and treatment groups (NPC) showed relatively higher alterations comparing to non-treated group (VEH, *p* = 0.65). However, no significant differences were observed between groups. [Fig. 3A] With respect to the PAT, BCAS mice (VEH: 92.1 ± 30.4) exhibited a significant reduction in escape latency than normal mice (sham: 244.6 ± 45.4, *p* = 0.004). However, NPC mice (252.0 ± 47.4) showed a significantly higher latency than non-treated mice (*p* = 0.001) [Fig. 3B] In the NORT, BCAS mice (VEH: 0.2 ± 0.07) exhibited a significant reduction in the discrimination index than normal mice (sham: 0.55 ± 0.02, *p* = 0.002). Instead, NPC mice (0.44 ± 0.05) showed higher discrimination indices than non-treated mice (VEH: 0.2 ± 0.07) with significance (*p* = 0.043). [Fig. 3C].

### IHC analyses

**Fig. 4:**
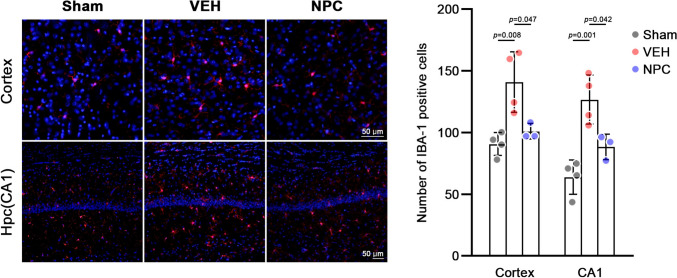
Immunohistochemistry (IHC) results of Iba-1 antibody in the cerebral cortex and hippocampus. Data are presented for three groups: Sham, VEH, and NPC. Statistical significance is indicated by *p*-values, with n.s. denoting non-significant differences (*p* > 0.05). Each data point represents an individual mouse, with error bars showing mean ± SEM

In the IHC analyses, we attempted to determine the inflammatory reactions using two antibodies: Iba-1 for microglia and GFAP for astrocyte activation. We observed a significant reduction in Iba-1-positive cells in the cerebral cortex of the NPC-treated mice than in the non-treated mice (VEH) [Fig. 4]. In the hippocampal section, mice treated with NPC (*p* = 0.042) showed significantly lower microglial activation than non-treated mice.

**Fig. 5:**
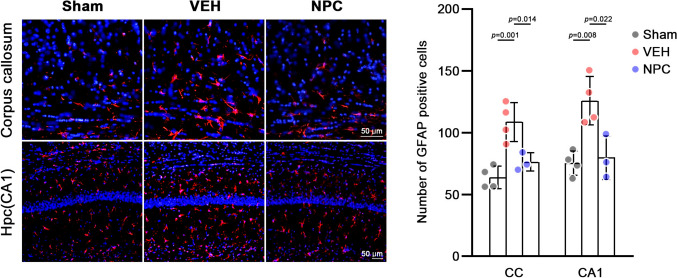
Immunohistochemistry (IHC) results of glial fibrillary acidic protein (GFAP) antibody in the cerebral cortex and hippocampus. Data are presented for three groups: Sham, VEH, and NPC. Statistical significance is indicated by *p*-values, with n.s. denoting non-significant differences (*p* > 0.05). Each data point represents an individual mouse, with error bars showing mean ± SEM

We also observed a significant reduction in GFAP-positive cells in the corpus callosum of the treated mice (NPC) than in the non-treated mice (VEH). In the cortical sections, only mice treated with NPC showed significantly lower astrocyte activation (Fig. 5; *p* = 0.022).

**Fig. 6:**
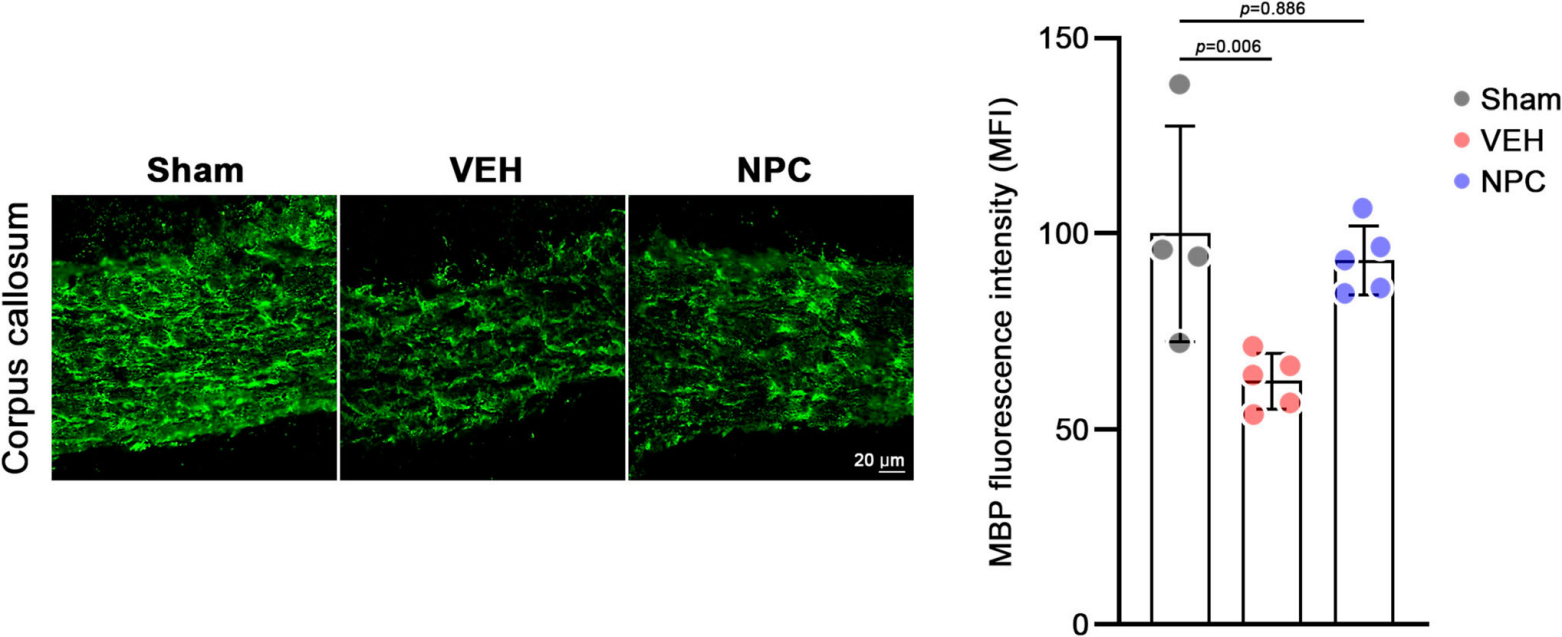
Immunohistochemistry (IHC) results of myelin basic protein (MBP) antibody in the corpus callosum. Data are presented for three groups: Sham, VEH, and NPC. Statistical significance is indicated by *p*-values, with n.s. denoting non-significant differences (*p* > 0.05). Each data point represents an individual mouse, with error bars showing mean ± SEM

Myelin was fluorescently stained with the MBP antibody, and considerably reduced or degenerated myelin was observed in BCAS mice (VEH), but not in NPC-treated mice. NPC-treated mice showed similar results to those of the sham group mice, which can be interpreted as vascular-compromised axonal injuries that might be regenerated with NPC injections. [Fig. 6].

## Discussion

In summary, PAT latency and discrimination index in the NORT increased significantly in the NPC group than in the VEH group. In addition, the alterations in YMT were not considerably higher in the NPC group than in the VEH group. IHC results revealed that the GFAP- and Iba-1-positive cells were remarkably lower in the NPC group than in the VEH group, and MBP density was higher in the NPC group. Based on our data, hiPSC-derived NPCs seem to have therapeutic potential in cerebral hypoperfusion of VaD mice; it improves the working memory of VaD animals by diminishing the inflammatory reaction and protecting them from demyelination. These findings suggest that hiPSC-derived NPCs may be a safe and eligible treatment option for VaD, and intravenous NPC administration may be a promising cell-based and patient-specific therapy for VaD.

As described above, VaD is an increasingly common diagnosis in the elderly population [1], and VaD incidence is gradually increasing such that it is definitively regarded as the second most prevalent type of dementia in epidemiological studies [2]. However, we do not have many treatment options and those are limited to the mechanism of encouraging neurotransmissions, prevention of secondary injury, or vasodilations [27]. Owing to their own damage tissue regeneration mechanism, cell replacement therapies for degenerative neurological disease are widely studied. However, most studies are limited to animal experiments and usually focus on other neurodegenerative diseases, such as Alzheimer’s and Parkinson’s disease or cerebral infarctions.

This might be due to the following reasons: (1) Clear VaD diagnosis is clinically difficult because VaD manifestations are somewhat broad. VaD is a general term that describes problems with reasoning, planning, judgment, memory, and other thought processes caused by brain damage from impaired blood flow to the brain (vascular disease). Cerebral vascular diseases include cerebral infarction, intracranial hemorrhage, congenital or hereditary vascular disease (Moyamoya disease), and arteriopathy-associated chronic ischemia. From a clinical perspective, VaD can be narrowed by subcortical (WM) ischemic changes (leukoaraiosis) due to a chronic vascular compromised status, since other vascular diseases usually have specific localized lesions and abrupt symptom onset [5].

(2) No representative (novel) animal models are available for VaD. Since VaD includes various disease entities, many vascular compromised animal models exist, including global or focal hypoperfusion models, embolic occlusion models, hypertensive arteriopathy models, and infarction models [28–30]. In this study, we selected chronic bilateral vascular compromised animal models receiving BCAS. In particular, the current BCAS model is a theoretically excellent VaD model for inducing chronic hypoperfused ischemia following axonal fiber demyelination in the WM. This model affects “global” subcortical WM lesions not expressed by the transgenic mouse model of cerebral autosomal dominant arteriopathy with subcortical infarcts and leukoencephalopathy (CADASIL) [31]. To our results, VaD model was successfully developed, which demonstrated poor neurobehavioral symptoms (YMT, PAT, and NORT), increased neuroinflammations (Iba-1, GFAP), and MBP degeneration.

Herein, we attempted to verify the efficacy of hiPSC-derived NPCs in VaD animal models. The products were prepared using our own protocols, which are registered in the preceding patent (10-2019-0071402). hiPSC-derived NPCs are multipotent stem cells that are capable of proliferation, self-renewal, and generation of new neurons, astrocytes, and oligodendrocytes [32]. Theoretically, the NPCs can migrate to injured tissues, likely due to inflammatory signals, demonstrating their potential to hone to the vasculature where the cells may confer neuroprotective effects in diseases characterized by inflamed vasculature, such as Parkinson’s disease, cerebral infarction, and VaD [33]. Because neurological deficits entail stem cell dysfunction due to “aging” as the common denominator [34], cell replacement therapy is an anti-aging treatment, at least in attenuating the cognitive impairments owing to aging. Functional recovery of the damaged and aging brain can be augmented by exogenous stem cell replacement or endogenous stem cell stimulation [35]. Exploiting the inflammatory pathway molecules that induce endogenous stem cells to secrete anti-inflammatory factors may also represent a robust strategy to treat inflammation-associated disorders, including VaD [36].

Our findings suggest that the therapeutic effects of intravenously administered NPCs in VaD are primarily mediated through paracrine mechanisms rather than direct cell integration. Unlike acute stroke or hemorrhage models where blood–brain barrier (BBB) disruption facilitates cell migration, VaD is characterized by chronic hypoperfusion with an intact BBB. This intact barrier presents a significant challenge for direct cell migration into the brain parenchyma [23]. Indeed, our PKH-26 staining [Supplementary Fig. 1] revealed minimal presence of administered cells in brain tissue, supporting this understanding. However, despite limited cell migration, we observed significant improvements in behavioral outcomes and histological markers, particularly in neuroinflammation and myelin integrity. These improvements likely result from the secretome of administered NPCs, including various bioactive factors that can cross the BBB and modulate the local microenvironment. This paracrine effect aligns with recent paradigm shifts in stem cell research, which increasingly recognize that therapeutic benefits often occur through secreted factors rather than direct cell replacement, especially in conditions where the BBB remains intact [21].

In South Korea, choline alfoscerate (= ChA) is widely prescribed as one of the primary pharmacological agents for treating VaD, with national health insurance coverage [37]. While its clinical efficacy is debated as some studies have reported modest improvements in cognitive function, ChA has demonstrated more consistent and significant effects in animal models of dementia [38]. ChA restores cholinergic transmission and improves cognitive deficits associated with cholinergic system impairment, which is a key feature of various forms of dementia, including VaD [39, 40]. Given these factors, we additionally designed a study with ChA as the comparator in this study to demonstrate the potential superiority of NPC therapy over an already recognized therapeutic option in both preclinical and clinical contexts. ChA (Daewoong Bio Co., Ltd., Gyeonggido, Korea) was administered intravenously at a dosage of 100 µL (5 mg/mL) to six mice (n = 6) for 15 days following BCAS surgery. The intravenous route was chosen to ensure consistent drug levels and bioavailability. [41, 42]

Our results [Supplementary Fig. 2] showed that ChA treatment produced moderate improvements in behavioral tests and inflammatory markers compared to the VEH group, though not as pronounced as the NPC group. Specifically, the ChA group showed improved discrimination index in NORT and reduced neuroinflammation markers (Iba-1 and GFAP-positive cells), but the improvements in learning ability (PAT) and myelin integrity (MBP) were less significant compared to NPC treatment.

This study has several limitations. First, the sample size was relatively small and the number of IHC markers assessed was limited. Second, the evaluation of neurological deficits was based solely on behavioral assessments. Third, we only observed axonal degeneration (MBP) in the corpus callosum, which bridges the bilateral hemispheres, and broad WM lesions of the corona radiata or the internal capsule were excluded. As leukoaraiosis of the WM is a pathognomonic finding of VaD in the human brain, efforts to evaluate both the corpus callosum and WM are necessary in the current experiment with mice. Additionally, while our study demonstrates the therapeutic potential of NPCs through behavioral assessments and inflammation reductions, it does not identify specific key molecules responsible for these effects. Comprehensive genomic and proteomic analyses are needed to uncover these molecular mechanisms, which could further elucidate the pathways involved in NPC-mediated therapy. Future studies should aim to explore these aspects to enhance our understanding of the therapeutic processes. Finally, our use of the micro-coil technique to induce chronic hypoperfusion may limit the immediate assessment of cerebral blood flow (CBF) changes during the acute phase, as significant alterations may not be evident until two weeks post-surgery. Thus, preoperative CBF measurements should be considered in future studies to better quantify ischemia across subjects.

In conclusion, hiPSC-derived NPCs appear to have therapeutic potential for cerebral hypoperfusion in VaD mice. It improved the working memory of VaD animals (PAT and NORT) by diminishing inflammatory reactions (Iba-1 and GFAP) and protecting against demyelination (MBP). These findings suggest that hiPSC-derived NPCs may be safe and eligible to treat VaD, and intravenous NPC administration may be a promising cell-based and patient-specific therapy for VaD.

## Supplementary Information

Below is the link to the electronic supplementary material.

Supplementary Data  (Original data)

Supplementary Fig 1.

Supplementary Fig 2.

Supplementary Figure Legends.

## Data Availability

Data supporting the findings of this study are not publicly available (except Supplementary Data uploaded). However, they are available from the authors upon reasonable request and with permission from the corresponding author (D-H Park).
