# Supplementary material for: Efficacy of Human-Induced Pluripotent Stem Cell-Derived Neural Progenitor Cell Replacement Therapy in a Vascular Dementia Animal Model
Source: Tissue Eng Regen Med. 2025 Feb 14;22(3):339–49. doi: 10.1007/s13770-025-00706-z (PMC11926306; doi:10.1007/s13770-025-00706-z)
Supplement: Supplementary file 1 — Supplementary Data (Original data) [file 13770_2025_706_MOESM1_ESM.pdf]

		이동경로
Sham	1	ABCBCAACBACACBABACBABABCABCABCABCA
	2	CABABCBCBCACABCABCBABCBACBABABCABCABCABC
	420	BACACBACBCBACBABCACBA
	421	ABACACABCACABCABCABCABCABCABCBCABCB
	422	BCACBABCABCACABABCABCACABCABCBACAB
VEH	1	BABACBCBACABABCBCBABACBABACBCBABCB
	2	ABCACACBABCABCAB
	3	CBACABABABABCACABCABBCBCAACBABABCBAB
	4	ACBABABCCABABCBABCACBCBACABACBACBA
	5	CACBABACBABBACABACBABABACACCBACBACABA
	6	BACBABABCABABCACBCACBACBCBACBACBABCBCBA CBCABA
NPC	1	ABABCACABCACBCABCABCBCABCABABCABACBC
	2	ACBABCACBABABACABCBCABACBACAACB
	3	BABABACBACBACBACBABCBCBACACBACBACBACBACBC
	4	ABABCABCABABABCBCABCBCACBCACBCBACBBACB
	5	CBACABABCBCABCBCBABCBABCACABCBCBCABCABCB
	6	BACABCBACBCBACBABABCBACBCABCBCACABCACBCA
	7	CBACABCACBABCBCBABCBABCABABCBC
Choline Alfoscerate	1	ACBCBACABCBACBACBACABCABCBCBCABAC
	2	BABABCABCABACBACBCACB
	3	ACBACBACBABACBCBACACABBCABABCACABCBCB
	3	CACBACABABCABCABACBACBABCABCBCABA
	4	BACABABCABCACABACBCABC
	5	CACBACACBCABABCACABBABCBACBBABCBCBABACBACACAB
	6	CABCACBBCABCBCBACACBACBABBCBA

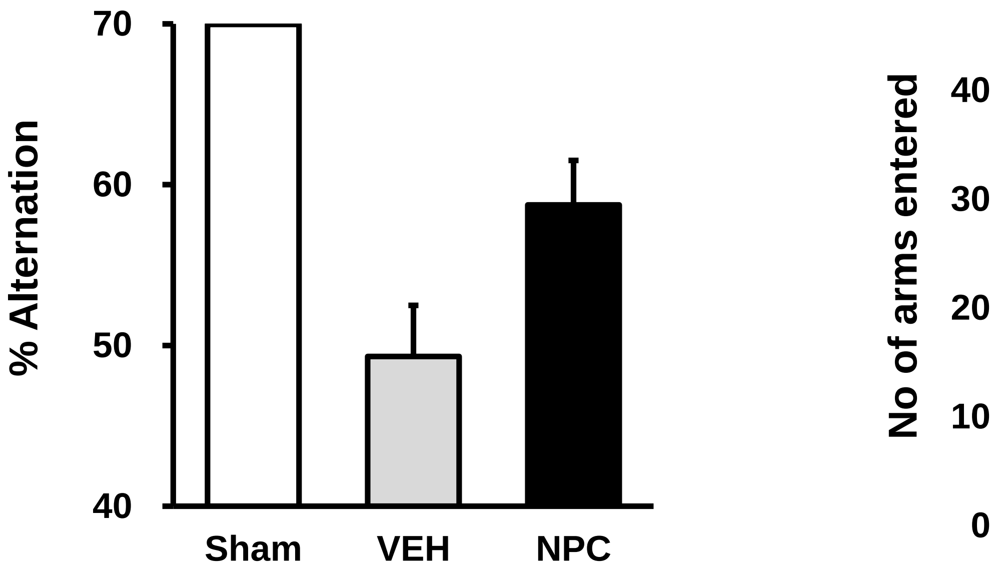

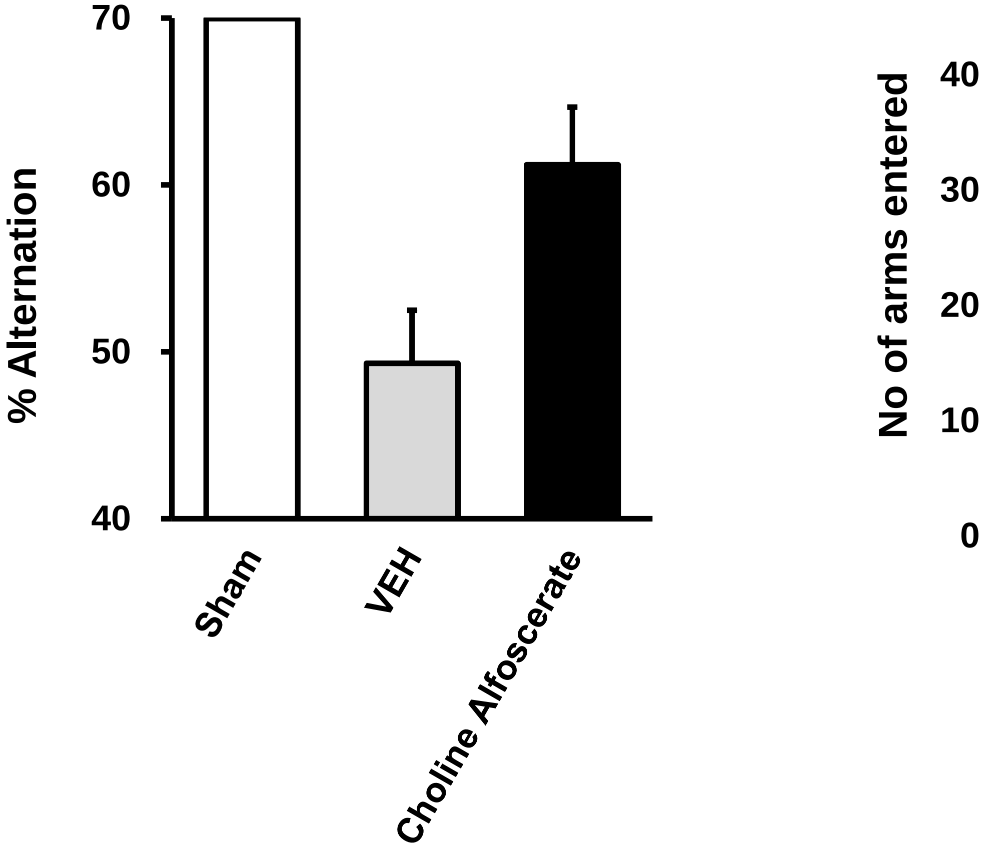

70
60
50
40
% Alternation
Sham
VEH
Choline Alfoscerate
No of arms entered
40
30
20
10
0

	Alternation (%)	No of arms entered	
0.66	65.6	34	
0.63	63.2	40	
0.68	68.4	21	
0.73	72.7	35	
0.69	68.8	34	
	Mean	67.7	32.8
	SE	#NAME?	3.5
0.44	43.8	34	
0.71	71.4	16	
0.41	41.2	36	
0.56	56.3	34	
0.49	48.6	37	
0.57	56.8	46	
	Mean	49.3	37.4
	SE	3.2	2.2
0.65	64.7	36	
0.55	55.2	31	
0.72	71.8	41	
0.53	52.8	38	
0.53	52.6	40	
0.61	60.5	40	
0.54	53.6	30	
	Mean	58.7	36.6
	SE	2.8	1.7
0.68	67.7	33	
0.68	68.4	21	
0.54	54.3	37	
0.71	71.0	33	
0.65	65.0	22	
0.47	46.5	45	
0.56	55.6	29	
			31.4
			3.2

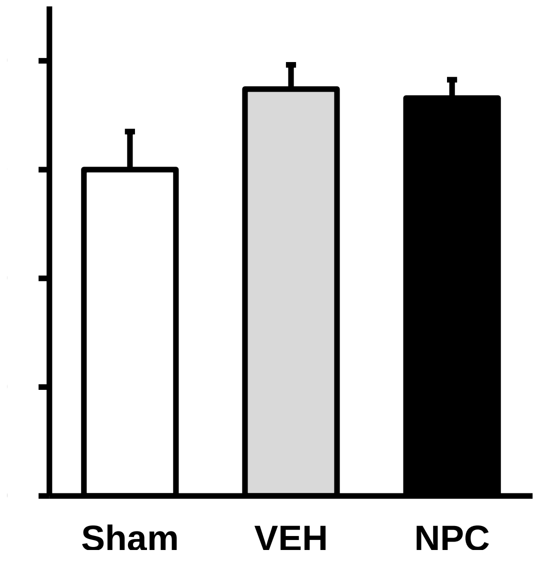

Alternation (%)

	Mean
Sham	70.0
VEH	49.3
NPC	58.7

	Mean
Sham	70.0
VEH	49.3
Choline Alfoscerate	61.2

정규성, 등분산 만족	(I) 그룹1
Scheffe	Sham
	VEH
	NPC

정규성 만족	
Scheffe	Sham
	VEH
	Choline alfoscerate
Bonferroni	Sham
	VEH
	Choline alfoscerate

KW 분석 ->bonferroni correction,

Sham vs. VEH

	cholYmaze
Mann-Whitney의 U	0.000
Wilcoxon의 W	15.000
Z	-2.611
근사 유의확률(양측)	.009
정확한 유의확률 [2*(단측 유의확률)]	.008[b]

VEH vs. Choline Alfo

	cholYmaze

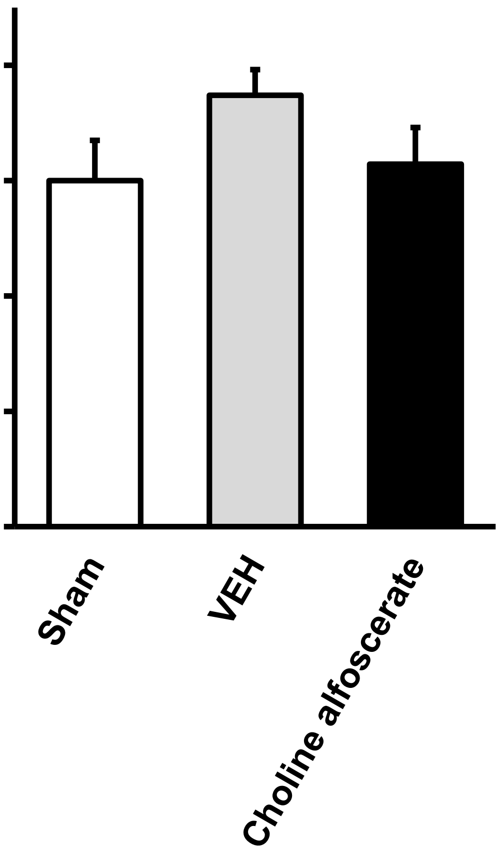

Mann-Whitney의 U	7.000
Wilcoxon의 W	22.000
Z	-1.705
근사 유의확률(양측)	.088
정확한 유의확률 [2*(단측 유의확률)]	.106[b]

Sham vs. Choline Alfo

	cholYmaze
Mann-Whitney의 U	9.500
Wilcoxon의 W	37.500
Z	-1.301
근사 유의확률(양측)	.193
정확한 유의확률 [2*(단측 유의확률)]	.202[b]

No of arms entered

SE		Mean	SE
1.1	Sham	30.0	3.5
3.2	VEH	37.4	2.2
2.8	NPC	36.6	1.7

SE		Mean	SE
1.1	Sham	30.0	3.5
3.2	VEH	37.4	2.2
3.5	Choline alfoscerate	31.4	3.2

(J) 그룹1	평균차(I-J)	표준오차	유의확률
VEH	18.4	4.05	**0.002**
NPC	8.997	3.749	0.090
Sham	-18.4	4.05	0.002
NPC	-9.403	3.749	0.074
Sham	-8.997	3.749	0.090
VEH	9.403	3.749	0.074

VEH	18.400*	4.637	.005
Choline alfoscerate	6.526	4.293	.343
Sham	−18.400*	4.637	.005
Choline alfoscerate	−11.874*	4.293	.047
Sham	−6.526	4.293	.343
VEH	11.874*	4.293	.047
VEH	18.400*	4.637	.004
Choline [illegible]	6.526	4.293	.452
Sham	−18.400*	4.637	.004
Choline alfoscerate	−11.874*	4.293	.045
Sham	−6.526	4.293	.452
VEH	11.874*	4.293	.045

p value <0.0167

PAT	no.	Training day	Testing day
		Latency (sec)	Latency (sec)
**Sham**	1	7.1	256.2
	2	3.8	198.6
	420	4.4	300
	421	3.1	270.8
	422-x	21.9	197.2
	mean	8.1	244.6
	sd	7.9	45.4
**VEH**	1	22.5	89.5
	2	17.1	64.8
	3	13.7	149.2
	4	15.8	81.6
	5	16.5	70.6
	6	11.1	96.9
	mean	16.1	92.1
	sd	3.8	30.4
**NPC**	1	13.9	78.5
	2	6.5	180.1
	3	10.4	227.4
	4	11.6	300
	5	18.4	300
	6	14..7	231.9
	7	15.4	272.6
	mean	12.5	252.0
	sd	4.2	47.4
**Choline Alfo**	1	10.6	62.5
	2	26.1	61.8
	3	16.4	112.9
	4	16.8	275.6
	5	7.5	60.7
	6	12.5	4.8

다중 비교

종속 변수: Ymaze
Scheffe

(I) 그룹		평균차(I-J)	표준오차	유의확률	95% 신뢰구간	
					하한값	상한값
Sham	VEH	15.840*	4.747	.028	1.37	30.31
	NPC	9.990	4.747	.251	-4.48	24.46
	Choline A	6.526	4.590	.578	-7.47	20.52
VEH	Sham	-15.840*	4.747	.028	-30.31	-1.37
	NPC	-5.850	4.526	.650	-19.65	7.95
	Choline A	-9.314	4.361	.240	-22.61	3.98
NPC	Sham	-9.990	4.747	.251	-24.46	4.48
	VEH	5.850	4.526	.650	-7.95	19.65
	Choline A	-3.464	4.361	.888	-16.76	9.83
Choline A	Sham	-6.526	4.590	.578	-20.52	7.47
	VEH	9.314	4.361	.240	-3.98	22.61
	NPC	3.464	4.361	.888	-9.83	16.76

*. 평균차는 0.05 수준에서 유의합니다.

종속 변수: P[illegible]
Scheffe

(I) 그룹
Sham
VEH
NPC
Choline A

*. 평균차는 [illegible]

다중 비교

종속 변수: Entry
Scheffe

(I) 그룹		평균차(I-J)	표준오차	유의확률	95% 신뢰구간	
					하한값	상한값
Sham	VEH	-4.367	4.026	.760	-16.70	7.96
	NPC	-3.867	4.026	.820	-16.20	8.46
	Choline A	1.633	4.026	.983	-10.70	13.96
VEH	Sham	4.367	4.026	.760	-7.96	16.70
	NPC	.500	3.838	.999	-11.26	12.26
	Choline A	6.000	3.838	.502	-5.76	17.76
NPC	Sham	3.867	4.026	.820	-8.46	16.20
	VEH	-.500	3.838	.999	-12.26	11.26
	Choline A	5.500	3.838	.573	-6.26	17.26
Choline A	Sham	-1.633	4.026	.983	-13.96	10.70
	VEH	-6.000	3.838	.502	-17.76	5.76
	NPC	-5.500	3.838	.573	-17.26	6.26

다중 비교

종속 변수: NOR
Scheffe

(I) 그룹	(J) 그룹	평균차(I-J)	표준오차	유의확률	95% 신뢰구간	
					하한값	상한값
Sham	VEH	152.460*	34.492	.004	46.26	258.66
	NPC	-7.440	34.492	.997	-113.64	98.76
	Choline A	129.860*	36.026	.018	18.94	240.78
VEH	Sham	-152.460*	34.492	.004	-258.66	-46.26
	NPC	-159.900*	32.887	.001	-261.16	-58.64
	Choline A	-22.600	34.492	.933	-128.80	83.60
NPC	Sham	7.440	34.492	.997	-98.76	113.64
	VEH	159.900*	32.887	.001	58.64	261.16
	Choline A	137.300*	34.492	.009	31.10	243.50
Choline A	Sham	-129.860*	36.026	.018	-240.78	-18.94
	VEH	22.600	34.492	.933	-83.60	128.80
	NPC	-137.300*	34.492	.009	-243.50	-31.10

*. 평균차는 0.05 수준에서 유의합니다.

다중 비교

평균차(I-J)	표준오차	유의확률	95% 신뢰구간 하한값	95% 신뢰구간 상한값
.3600*	.0849	.005	.100	.620
.1267	.0849	.540	-.133	.387
.1767	.0849	.262	-.083	.437
-.3600*	.0849	.005	-.620	-.100
-.2333	.0810	.070	-.481	.015
-.1833	.0810	.199	-.431	.065
-.1267	.0849	.540	-.387	.133
.2333	.0810	.070	-.015	.481
.0500	.0810	.943	-.198	.298
-.1767	.0849	.262	-.437	.083
.1833	.0810	.199	-.065	.431
-.0500	.0810	.943	-.298	.198

유의합니다.
