# Supplementary figures and images for: Efficacy of Human-Induced Pluripotent Stem Cell-Derived Neural Progenitor Cell Replacement Therapy in a Vascular Dementia Animal Model
Source: Tissue Eng Regen Med. 2025 Feb 14;22(3):339–49. doi: 10.1007/s13770-025-00706-z (PMC11926306; doi:10.1007/s13770-025-00706-z)

**A**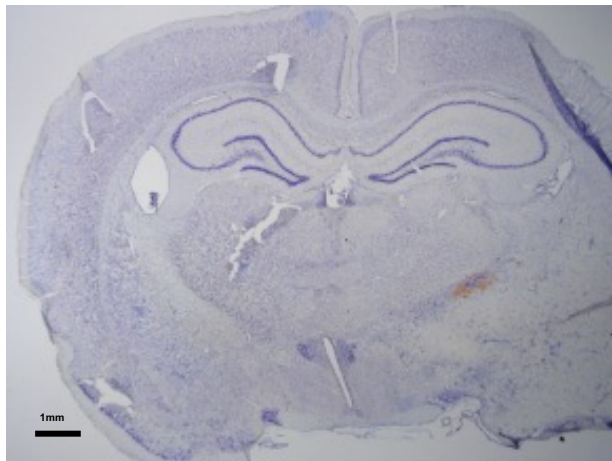**B**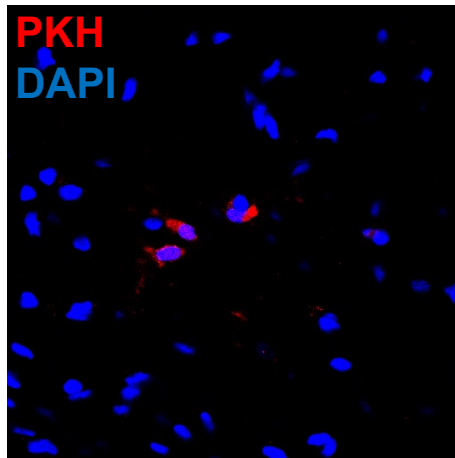**Cerebral cortex****C**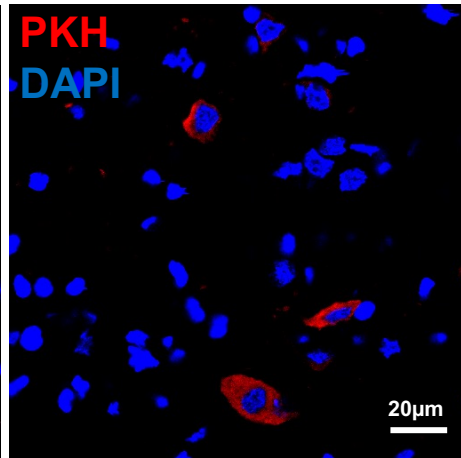**Hippocampus**

Supplement: Supplementary file 2 — Supplementary Fig 1. [file 13770_2025_706_MOESM2_ESM.pdf]
