# Supplementary material for: Efficacy of Human-Induced Pluripotent Stem Cell-Derived Neural Progenitor Cell Replacement Therapy in a Vascular Dementia Animal Model
Source: Tissue Eng Regen Med. 2025 Feb 14;22(3):339–49. doi: 10.1007/s13770-025-00706-z (PMC11926306; doi:10.1007/s13770-025-00706-z)
Supplement: Supplementary file 3 — Supplementary Fig 2. [file 13770_2025_706_MOESM3_ESM.pdf]

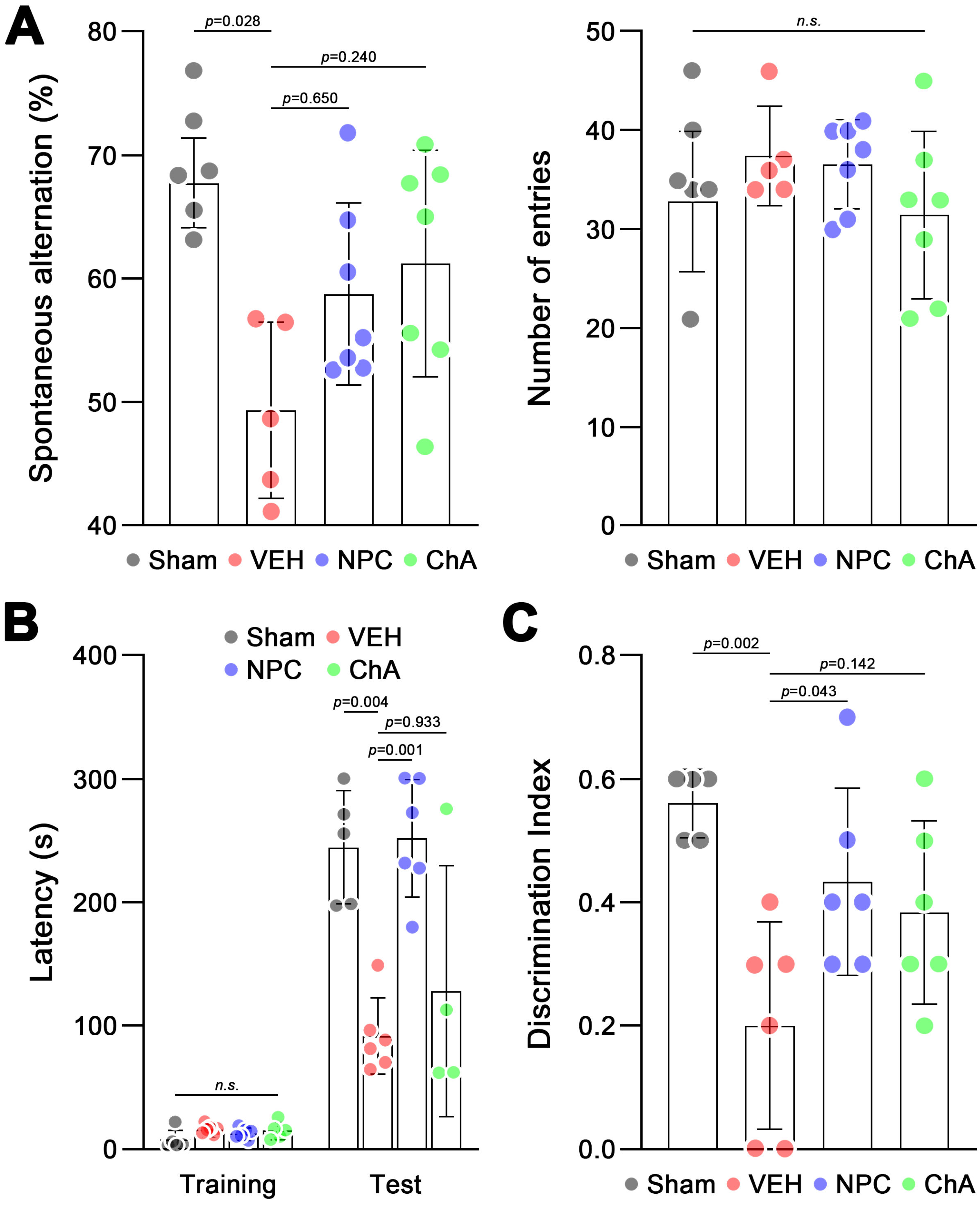

Results of neurobehavioral tests assessing cognitive function in mice

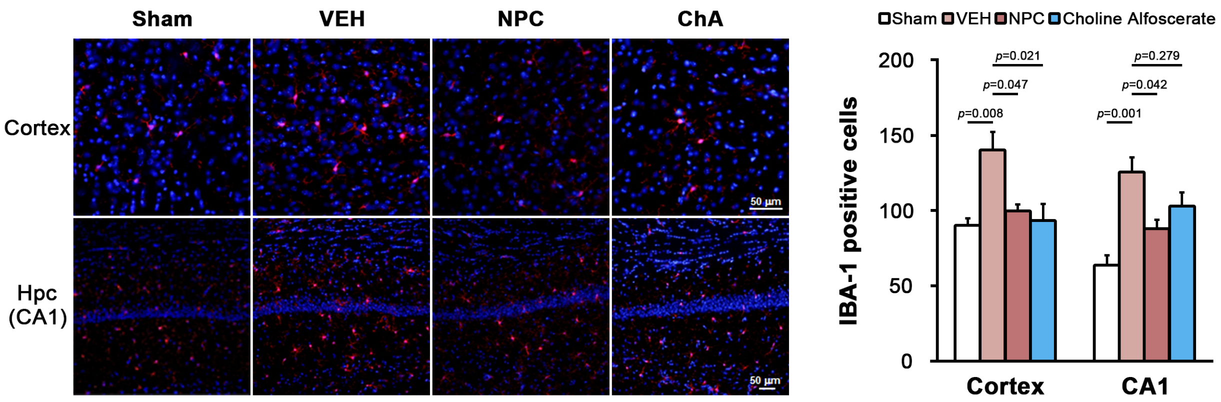

Immunohistochemistry (IHC) results of Iba-1 antibody in the cerebral cortex and hippocampus

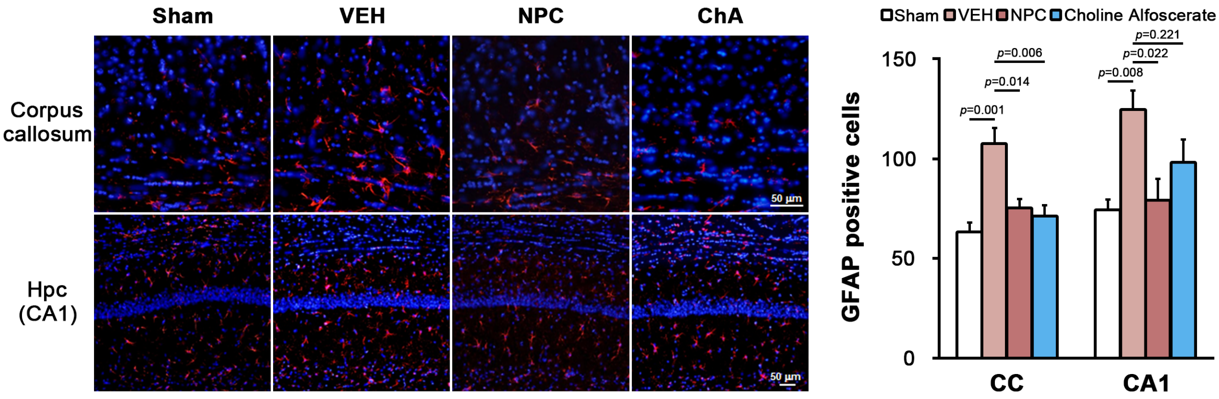

Immunohistochemistry (IHC) results of glial fibrillary acidic protein (GFAP) antibody in the cerebral cortex and hippocampus.

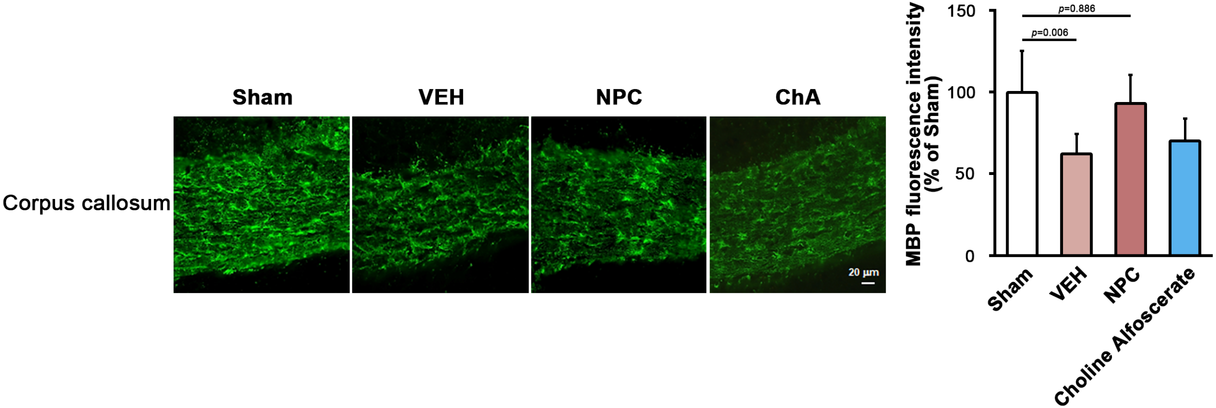

Immunohistochemistry (IHC) results of myelin basic protein (MBP) antibody in the corpus callosum.
