# Supplementary material for: Efficacy of Human-Induced Pluripotent Stem Cell-Derived Neural Progenitor Cell Replacement Therapy in a Vascular Dementia Animal Model
Source: Tissue Eng Regen Med. 2025 Feb 14;22(3):339–49. doi: 10.1007/s13770-025-00706-z (PMC11926306; doi:10.1007/s13770-025-00706-z)
Supplement: Supplementary file 4 — Supplementary Figure Legends. [file 13770_2025_706_MOESM4_ESM.docx]

**Sup Fig. 1**

PKH-26 labeled cell tracking in brain sections after intravenous NPC administration. (A) Representative coronal section showing the overall brain structure with regions of interest (black squares) in the cerebral cortex and hippocampus. (B) High magnification images of PKH-26 (red) labeled cells with DAPI (blue) nuclear counterstain in the cerebral cortex. (C) High magnification images of PKH-26 (red) labeled cells with DAPI (blue) nuclear counterstain in the hippocampus. M2 marker staining is shown for comparison. Very few PKH-26 positive cells were detected in both regions, suggesting minimal migration of intravenously administered NPCs across the blood-brain barrier. Scale bars: 1mm (A), 20μm (B, C).

**Sup Fig. 2**

Comprehensive analysis of behavioral tests and immunohistochemistry results including ChA group. (1) Results of neurobehavioral tests assessing cognitive function in mice: Y-maze test (YMT) measuring spontaneous alternation percentage for spatial working memory; Passive avoidance test (PAT) showing latency times for learning and memory retention; Novel object recognition test (NORT) displaying the discrimination index for recognition memory. (2) Immunohistochemistry (IHC) results of Iba-1 antibody in the cerebral cortex and hippocampus, showing microglial activation. (3) IHC results of glial fibrillary acidic protein (GFAP) antibody in the cerebral cortex and hippocampus, demonstrating astrocyte activation. (4) IHC results of myelin basic protein (MBP) antibody in the corpus callosum, indicating myelin integrity. Data are presented for all experimental groups: Sham, VEH, NPC, and ChA. Statistical significance is indicated by p-values, with n.s. denoting non-significant differences (p>0.05). Each data point represents an individual mouse, with error bars showing mean ± SEM. Scale bars = 50 μm.
